# High WHSC1L1 Expression Reduces Survival Rates in Operated Breast Cancer Patients with Decreased CD8+ T Cells: Machine Learning Approach

**DOI:** 10.3390/jpm11070636

**Published:** 2021-07-05

**Authors:** Hyung-Suk Kim, Kyueng-Whan Min, Dong-Hoon Kim, Byoung-Kwan Son, Mi-Jung Kwon, Sang-Mo Hong

**Affiliations:** 1 Division of Breast Surgery, Department of Surgery, Hanyang University Guri Hospital, Hanyang University College of Medicine, Guri 15588, Korea; hyung6960@naver.com; 2 Department of Pathology, Hanyang University Guri Hospital, Hanyang University College of Medicine, Guri 15588, Korea; 3 Department of Pathology, Kangbuk Samsung Hospital, Sungkyunkwan University School of Medicine, 29 Saemunanro, Seoul 03181, Korea; 4 Uijeongbu Eulji Medical Center, Department of Internal Medicine, Eulji University School of Medicine, Daejeon 34824, Korea; sbk1026@eulji.ac.kr; 5 Department of Pathology, Hallym University Sacred Heart Hospital, Hallym University College of Medicine, Anyang 24252, Korea; mulank@hanmail.net; 6 Division of Endocrinology, Department of Internal Medicine, Hanyang University Guri Hospital, Hanyang University College of Medicine, Guri 15588, Korea; lanugo@hanyang.ac.kr

**Keywords:** WHSC1L1, breast neoplasm, prognosis, tumor infiltrating lymphocyte, PD-L1

## Abstract

Nuclear receptor-binding SET domain protein (NSD), a histone methyltransferase, is known to play an important role in cancer pathogenesis. The WHSC1L1 (Wolf-Hirschhorn syndrome candidate 1-like 1) gene, encoding NSD3, is highly expressed in breast cancer, but its role in the development of breast cancer is still unknown. The purpose of this study was to analyze the survival rates and immune responses of breast cancer patients with high WHSC1L1 expression and to validate the results using gradient boosting machine (GBM) in breast cancer. We investigated the clinicopathologic parameters, proportions of immune cells, pathway networks and in vitro drug responses according to WHSC1L1 expression in 456, 1500 and 776 breast cancer patients from the Hanyang University Guri Hospital, METABRIC and TCGA, respectively. High WHSC1L1 expression was associated with poor prognosis, decreased CD8+ T cells and high CD274 expression (encoding PD-L1). In the pathway networks, WHSC1L1 was indirectly linked to the regulation of the lymphocyte apoptotic process. The GBM model with WHSC1L1 showed improved prognostic performance compared with the model without WHSC1L1. We found that VX-11e, CZC24832, LY2109761, oxaliplatin and erlotinib were effective in inhibiting breast cancer cell lines with high WHSC1L1 expression. High WHSC1L1 expression could play potential roles in the progression of breast cancer and targeting WHSC1L1 could be a potential strategy for the treatment of breast cancer.

## 1. Introduction

Epigenetic modifications of histones, including acetylation, methylation, phosphorylation and ubiquitination, are known to play an important role in many cellular events linked to development and disease pathogenesis, including malignancy [1,2]. Previous studies investigated the aberrant expression of histone methylation and histone methyltransferases that regulate the epigenetic modification of histones and are closely associated with various cancers [3]. Therefore, targeting histone methyltransferase (HMTase) overexpression is an important part of the epigenetic treatment of cancers. The nuclear receptor-binding SET domain protein (NSD) family of HMTases is composed of NSD1, NSD2 and NSD3. These proteins are known to regulate chromatin integrity and gene expression primarily through the methylation of lysine 36 of histone H3 (H3K36), which is known as an indicator of transcriptional elongation [4,5]. Therefore, NSD family carcinogenic proteins could affect cell proliferation and cancer progression.

NSD3 is located on the chromosome in the 8p11-p12 locus known as Wolf-Hirschhorn syndrome candidate 1-like 1 (WHSC1L1), which is amplified in breast cancer cell lines [6]. In previous studies, high WHSC1L1 expression has been shown to be common in lung cancer and pancreatic cancer [7,8]. Other studies have shown that WHSC1L1 plays roles as an oncogene and a transforming gene, driving the development and progression of breast cancer [9,10]. In studies of breast cancer, high WHSC1L1 expression was associated with a poor prognosis [11,12]. Nevertheless, the biological functions of WHSC1L1 in the carcinogenesis of breast cancer are not well understood.

Cancer cell survival could depend on the interactions between cancer cells and immune cells that make up the tumor microenvironment (TME). The TME plays an important role in cancer progression and the response to treatment, thus affecting the patient’s outcome. Marked lymphocytic infiltrates, known as tumor-infiltrating lymphocytes (TILs), at the invasive front of the tumor could play a pivotal role in anticancer immunity and be beneficial prognostic factors in various cancers. Cytotoxic T lymphocytes (CTLs) that express CD8 on the cell surface play a major role in anticancer immunity [13]. The identification of different types of immune cells in the TME helps predict cancer prognosis [14]. Previous studies have reported an association between NSD1 and immune cells, but the association between WHSC1L1 and immune cells has never been reported to the best of our knowledge [15].

In this study, we assessed the clinicopathological parameters and survival rate according to WHSC1L1 expression in breast cancer cohorts from Hanyang University Guri Hospital (HYGH), Molecular Taxonomy of Breast Cancer International Consortium (METABRIC) and The Cancer Genome Atlas (TCGA) database [16]. We analyzed the effect of WHSC1L1 on the survival of breast cancer patients using the gradient boosting machine (GBM) algorithm [17]. In addition, we investigated gene sets related to WHSC1L1 using a pathway-based network [18,19]. Anticancer immune responses were analyzed by assessing the distributions of CD8+ T cells and CD4+ T cells. Using the Genomics of Drug Sensitivity in Cancer (GDSC) database as an in vitro drug screening platform, we found sensitive drugs in breast cancer cell lines with high WHSC1L1 expression [20,21].

## 2. Materials and Methods

### 2.1. Patient Selection

This study enrolled 456 invasive ductal carcinoma (IDC) patients with tissue samples obtained at HYGH in Korea between 2005 and 2015. The Reporting Recommendations for Tumor Marker Prognostic Studies (REMARK) criteria were followed throughout this study [22]. The inclusion criteria were as follows: (1) stage I–III breast cancer patients who underwent curative surgery; (2) patients who did not receive neoadjuvant chemotherapy; and (3) patients with available paraffin blocks of tumor tissues or complete clinical outcome data and follow-up data. We assessed the survival rate and clinicopathologic characteristics of the tumors, including age, T stage, N stage, histopathological grade, lymphatic invasion, vascular invasion, perineural invasion, hormonal receptors, human epidermal growth factor receptor 2 (HER2) status, Ki-67, P53, and anti-programmed death-ligand 1 (PD-L1) and patient follow-up information.

### 2.2. Tissue Microarray Construction and Immunohistochemistry in Our Cohort

**Figure 1:**
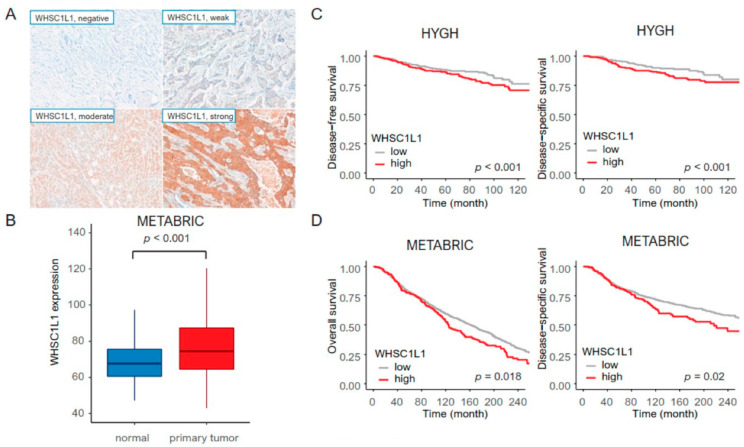
(**A**) The staining intensity was scored as negative (top left), weak (top right), moderate (bottom left), or strong (bottom right) from HYGH cohort (original magnification ×200). (**B**) METABRIC: High WHSC1L1 expression in primary tumors compared to that in normal tissues. (**C**) HYGH cohort: High WHSC1L1 expression was associated with poor disease-free survival (average survival time: high WHSC1L1 expression group, 67.2 months; low WHSC1L1 expression group, 87.6 months) (median survival time: high WHSC1L1 expression group, 63.5 months; low WHSC1L1 expression group, 95 months) and disease-specific survival (average survival time: high WHSC1L1 expression group, 74.6 months; low WHSC1L1 expression group, 95.3 months) (median survival time: high WHSC1L1 expression group, 71.5 months; low WHSC1L1 expression group, 98 months) (*p* < 0.001 and 0.001, respectively). (**D**) METABRIC: High WHSC1L1 expression was associated with poor overall survival (average survival time: high WHSC1L1 expression group, 123.8 months; low WHSC1L1 expression group, 125.3 months) (median survival time: high WHSC1L1 expression group, 116.4 months; low WHSC1L1 expression group, 115.5 months) and disease-specific survival (average survival time: high WHSC1L1 expression group, 126.3 months; low WHSC1L1 expression group, 136.1 months) (median survival time: high WHSC1L1 expression group, 117.2 months; low WHSC1L1 expression group, 119.5 months) (*p* = 0.018 and 0.02, respectively).

In HYGH samples, tissue microarray (TMA) blocks were assembled using a tissue array instrument (AccuMax Array; ISU ABXIS Co., Ltd., Seoul, Korea). We used duplicate 3 mm diameter tissue cores (tumor components in a tissue core > 70%) from each donor block. Four-micrometer sections were cut from the TMA blocks using routine techniques. Immunostaining for WHSC1L1 (1:100, 11345-1-AP; Proteintech, Manchester, UK), estrogen receptor (ER) (1:200, Lab Vision Corporation, Fremont, CA, USA), progesterone receptor (PR) (1:200, Dako, Glostrup, Denmark), HER2 (1:1, Ventana Medical Systems Tucson, Oro Valley, AZ, USA), anti-CD8 (clone 4B11, Leica Biosystems, Newcastle, UK), anti-CD4 (clone 4B12, Leica Biosystems, Newcastle, UK), P53 (1:5000, Cell Marque, Hot Springs, AR, USA) and Ki67 (1:200; MIB-1, Dako, Glostrup, Denmark) was performed using the Bond Polymer Refine Detection System (Leica Biosystems Newcastle Ltd., Newcastle, UK) according to the manufacturer’s instructions and the Dako Autostainer Universal Staining System (Dako, Carpinteria, CA, USA) with the ChemMate DAKO EnVision™ Detection Kit (Dako) [23]. PD-L1 (clone SP142, Ventana Medical Systems, Roche, Tucson, AZ, USA) staining was performed. The intensity of immunostaining was recorded as follows: 0 (no staining), 1 (weak staining), 2 (moderate staining), and 3 (strong staining) (Figure 1A). The proportion of staining was graded as follows: 0 (0–5%), 1 (6–25%), 2 (26–50%), 3 (51–75%), and 4 (>75%). The immunoreactive score (IRS) was calculated (intensity × proportion), and WHSC1L1 expression was determined to be either low (IRS ≤ 3) or high (IRS > 3).

### 2.3. Analysis Based on the METABRIC Database and TCGA Database

We obtained 1500 IDC cases from the METABRIC database with gene data (cDNA microarray profiling, Illumina HT-12 v3 platform) (https://www.cbioportal.org/) (accessed on 1 June 2021) [24]. The microarray from METABRIC (log-transformed data) was assessed, and WHSC1L1 expression was determined to be either low (log-scale scores < 7.14402497) or high (log-scale scores > 7.14402497). WHSC1L1 expression, overall survival (OS) and disease-specific survival (DSS) were extracted using the R package (http://www.r-project.org/) (accessed on 1 June 2021). Normal samples and tumor samples with missing data were excluded from the analysis.

We obtained 776 IDC cases with RNA-Seq data from the TCGA database [16]. The RNA-Seq data from TCGA was assessed, and WHSC1L1 expression was determined to be either low (scores < 1472.755) or high (scores > 1472.755). We applied in silico cytometry known as CIBERSORT (https://cibersort.stanford.edu/) (accessed on 1 June 2021) to determine the proportions of 22 subsets of immune cells using 547 genes [25]. For grouping of networks based on functionally enriched Gene Ontology (GO) terms and pathways, pathway network analyses were visualized using Cytoscape software (version 3.8.2). We observed which genes had the closest relationship with high WHSC1L1 expression using the kappa value and elucidated the functionally grouped Gene Ontology and pathway annotation networks using the ClueGO application (version 2.5.6), an app for Gene Ontology analysis [18,19].

### 2.4. Machine Learning Algorithm for Validation

We integrated WHSC1L1 with clinical risk factors (T stage, N stage, histological grade, lymphatic invasion, perineural invasion and ER) to construct prognostic models for survival prediction by applying machine learning (ML) algorithms in 456 cases (HYGH) (randomization: training set, 70%; validation set, 30%). A learning algorithm was independently applied to select and combine multiple covariates from GBM based on multivariate Gaussian models. In this step, the ‘‘forward” search method, which initiates on a prototype set and selects a feature if and only if the addition of the feature could increase the performance of the prognostic model, was adopted to select optimal features sequentially. The hyperparameters of the ML algorithms, such as the learning rate in GBM, were optimized for each combination of selected covariates and learning algorithm by grid search cross-validation through a predefined range. We searched 81 models with varying learning rates and tree depths. The final optimal models were trained based on the selected covariates and the optimized hyperparameters [17]. To explore the performance outcomes of the GBM method, the receiver operator characteristic (ROC) curve was used.

### 2.5. GDSC Database

We analyzed the relationship between anticancer drug sensitivity and WHSC1L1 expression based on the Genomics of Drug Sensitivity in Cancer (GDSC version 2.0) dataset, which contains data on the drug responses of approximately 19 breast cancer cell lines to 172 anticancer drugs [26]. We measured anticancer drug sensitivity in 19 breast cancer cell lines with the natural log-half-maximal inhibitory concentration (LN IC50). A drug was identified as an effective drug when the calculated LN IC50 value was decreased in cell lines with high WHSC1L1 expression and increased in those with low NSD3 expression, i.e., when an inverse correlation was observed. Pearson’s correlation and Student’s *t*-test were used to assess the comparisons between the LN IC50 values and WHSC1L1 expression [20,21].

### 2.6. Statistical Analysis

Correlations between several clinicopathological variables and WHSC1L1 expression were analyzed using the χ^2^ test. Student’s *t*-test and Pearson’s correlation were used to examine the differences between continuous variables. Disease-free survival (DFS) was defined as the time from the date of diagnosis to recurrence/new distant metastasis, and DSS was defined as the time from the date of diagnosis to cancer-related death. OS was defined as the time from the date of diagnosis to all-cause death. The Kaplan–Meier method was used to determine the probability of survival, and survival rates were compared using the log-rank test and Cox regression analyses. A two-tailed *p*-value of <0.05 was considered statistically significant. All data were analyzed using R software packages and SPSS statistics (version 25.0, SPSS Inc., Chicago, IL, USA).

## 3. Results

### 3.1. Clinicopathological Parameters and Survival Rate

We investigated a total of 1956 patients with WHSC1L1 expression data and survival data in the HYGH cohort and METABRIC. In the HYGH cohort, high WHSC1L1 expression was related to ER negativity and PR negativity (*p* = 0.04 and 0.017, respectively). High WHSC1L1 expression was frequently observed in tumors with PD-L1 negativity, high p53 expression and high Ki-67 (*p* = 0.001, 0.005 and <0.001, respectively) (Table 1). In METABRIC, WHSC1L1 expression was increased in primary tumors compared to normal tissues (*p* = 0.046) (Figure 1B).

In the HYGH cohort, patients with high WHSC1L1 expression had significantly worse DFS and DSS than those with low WHSC1L1 expression (high WHSC1L1 expression group, 354 patients; low WHSC1L1 expression group, 102 patients) (all *p* < 0.001) (Figure 1C). After adjustment for confounders, such as T stage, N stage, histological grade, lymphatic invasion, perineural invasion and ER, the significance remained (Table 2). Regarding molecular subtypes, such as luminal A, luminal B, HER2, and triple-negative, high WHSC1L1 expression was associated with short DSS and DFS in the luminal A (*p* < 0.001 and 0.001, respectively) and HER2 subtypes (*p* = 0.031 and 0.01, respectively).

In METABRIC, we investigated 1500 IDC patients to validate the relationship between WHSC1L1 and survival. High WHSC1L1 expression was significantly correlated with poor DSS and OS (high WHSC1L1 expression group, 168 patients; low WHSC1L1 expression group, 1332 patients) (*p* = 0.02 and *p* = 0.018, respectively) compared to low WHSC1L1 expression (Figure 1D).

### 3.2. Anticancer Immune Response and Pathway Network Analysis

**Figure 2:**
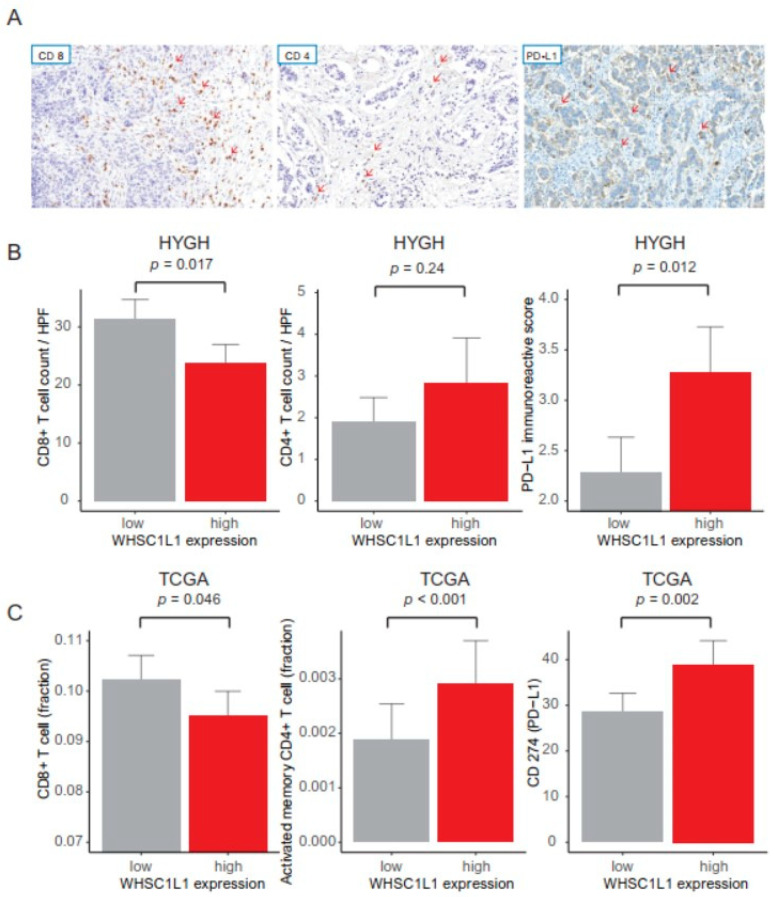
(**A**) Representative microphotographs showing CD8+ T cells, CD4+ T cells and PD-L1 expression from HYGH cohort (red arrows, positive stain (brown color)). (**B**) Bar plot of CD8+ T cell count, CD4+ T cell count per high-power field (HPF, ×400) and PD-L1 expression in HYGH cells. (**C**) Bar plot of CD8+ T cell fraction, activated memory CD4+ T cell fraction and CD274 (encoding PD-L1) expression in TCGA.

**Figure 3:**
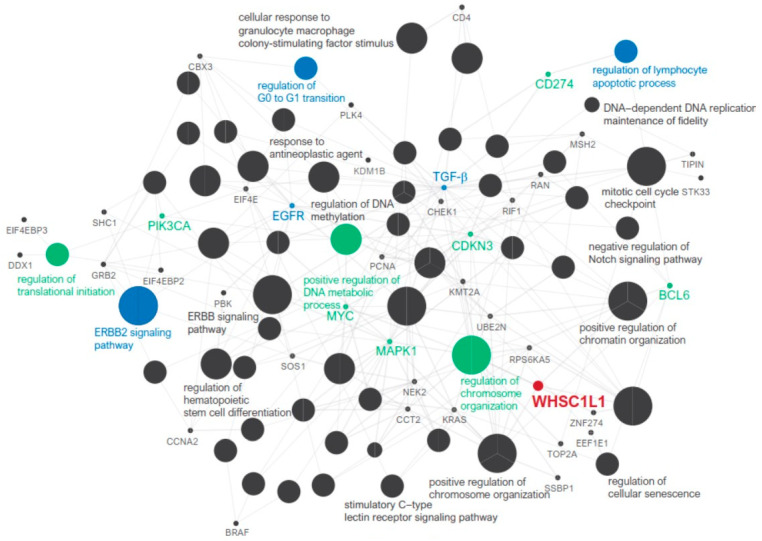
Functionally grouped networks are linked to their biological function, and only the most significant term of the group is labeled.

We analyzed the relationship between WHSC1L1 expression and immune cells using the HYGH and TCGA cohorts. In the HYGH cohort, high WHSC1L1 expression was associated with decreased CD8+ T cell count, increased CD4+ T cell count and high PD-L1 expression (*p* = 0.017, 0.024 and 0.012, respectively) (Figure 2A,B). In the TCGA cohort, high WHSC1L1 expression was also correlated with a low fraction of CD8+ T cells, a high fraction of CD4+ T cells and high CD274 (encoding PD-L1) expression (*p* = 0.046, < 0.001 and = 0.002, respectively) (Figure 2C). In pathway network analysis, WHSC1L1 was linked to the regulation of translation initiation, ERBB2 signaling pathway, positive regulation of the DNA metabolic process, regulation of chromosome organization and regulation of the lymphocyte apoptotic process (Figure 3).

### 3.3. Machine Learning and Drug Screening

**Figure 4:**
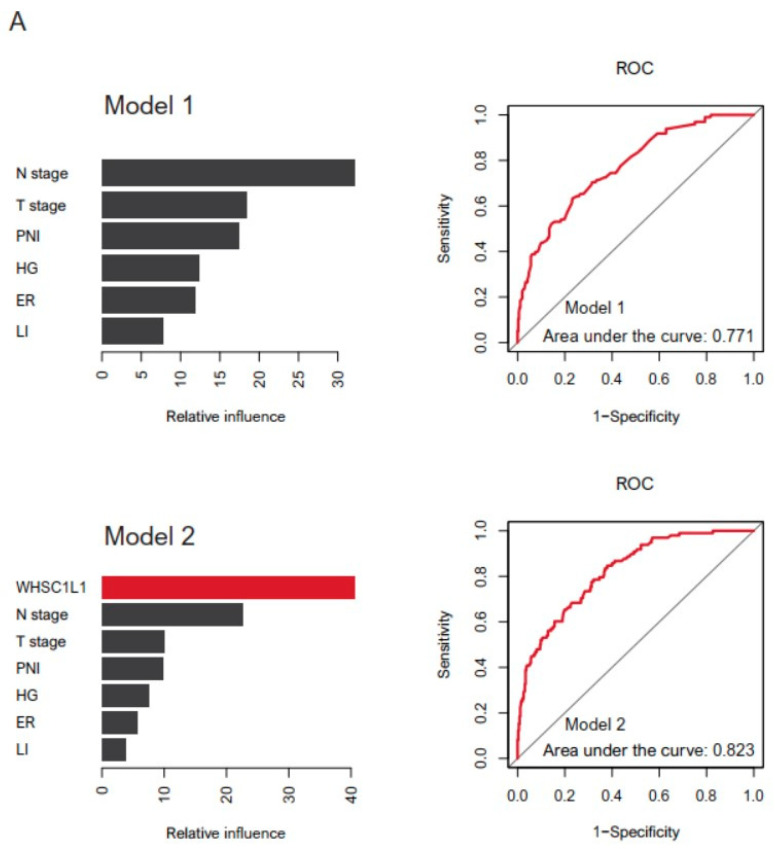

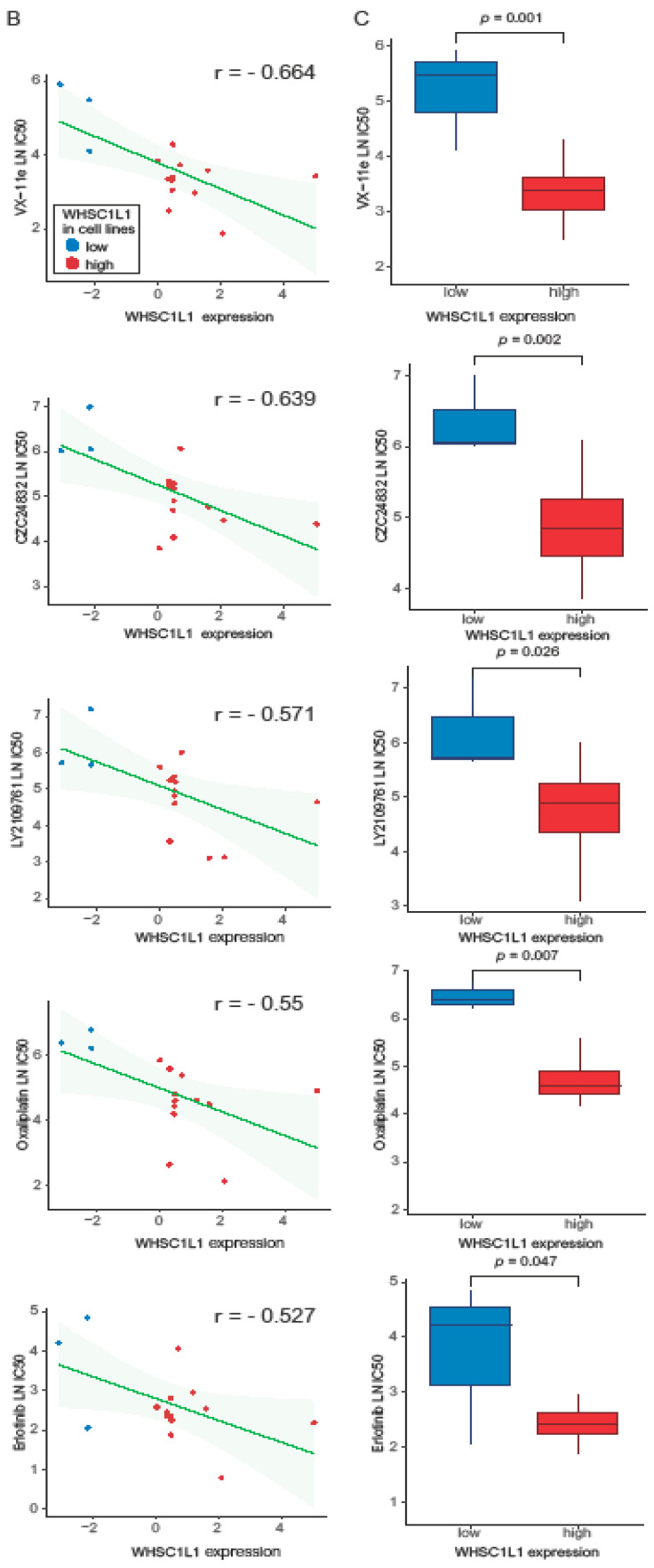
(**A**) We used the supervised machine learning model (gradient boosting machine) for prognosis prediction. Covariates were included as confounding factors (Model 1 (T stage, N stage, perineural invasion (PNI), histological grade (HG), estrogen receptor (ER) and lymphatic invasion (LI)) vs. Model 2 (WHSC1L1, T stage, N stage, PNI, HG, ER and LI)), and their relative importance was assessed using survival analysis. A receiver operator characteristic curve for GBM was used based on a multivariate Gaussian model. (**B**) Pearson’s correlation analysis showing the natural log of the half-maximal inhibitory concentration (LN IC50) values of VX-11e, CZC24832, LY2109761, oxaliplatin and erlotinib in breast cancer cells (blue, low WHSC1L1 expression; red, high WHSC1L1 expression). (**C**) Bar plot showing the LN IC50 values of VX-11e, CZC24832, LY2109761, oxaliplatin and erlotinib in breast cancer cells with low (blue) and high (red) WHSC1L1 expression (*p* = 0.001, 0.002, 0.026, 0.007 and 0.047, respectively) (error bars: standard errors of the mean).

We compared the performance of the two GBM models in predicting survival rates (Model 1 (T stage, N stage, histological grade, lymphatic invasion, perineural invasion, and ER) vs. Model 2 (WHSC1L1, T stage, N stage, histological grade, lymphatic invasion, perineural invasion, and ER)). ROC curves were generated (area under the curve: Model 1, 0.771; Model 2, 0.823). We found that the GBM algorithm performed the best, while the addition of WHSC1L1 to the prediction model improved the prognostic performance (Figure 4A).

In the GDSC database, we analyzed the drug sensitivity of 50 breast cancer cell lines according to WHSC1L1 expression. We found five anticancer drugs that most effectively reduced the growth of breast cancer cells with high WHSC1L1 expression: VX-11e, CZC24832, LY2109761, oxaliplatin and erlotinib (Figure 4B).

## 4. Discussion

This study found that WHSC1L1 was significantly overexpressed in breast cancer tissue compared to normal breast tissue. Higher WHSC1L1 expression was associated with worse DFS and DSS in breast cancer. To validate our results, we compared the relationship between the survival rate and WHSC1L1 in METABRIC. In this study, the inclusion of WHSC1L1 in the machine learning model increased the accuracy of predicting the survival rate. GBM, a type of machine learning, has the advantage of processing large amounts of predictors through simple prediction algorithms and combining the results in a non-linear and interactive way, which can improve the accuracy of predictions [17]. Therefore, our findings suggest that WHSC1L1 expression plays an important role in the development and progression of breast cancer as well as epigenetic regulation; thus, it is expected to contribute to effective treatments for breast cancer [27,28,29,30].

WHSC1L1, a histone methyltransferase, is an important driving oncogene of the amplification of 8p11-12 in breast cancer and is an epigenetic marker that regulates cell growth and differentiation [31,32,33,34]. WHSC1L1 expression is significantly elevated in various malignant tumors, such as breast cancer, bladder cancer, osteosarcoma, head and neck cancer, and colorectal cancer [7,35,36,37,38]. A previous study found that high WHSC1L1 expression was related to a high Ki-67 index and poor prognosis in breast cancer [5,31]. These results are consistent with our results.

The TME is composed of immune cells, fibroblasts, satellite cells, and blood vessels or lymphatic vessels. These factors play a pivotal role in tumor progression, treatment response, and clinical outcomes [13]. In the TME, cytotoxic T lymphocytes can induce the apoptosis of target cells through the cancer-immunity cycle [39]. In our study, high WHSC1L1 expression was related to decreased CD8+ T cell counts and high PD-L1 expression. We applied in silico flow cytometry to TCGA data and found that high WHSC1L1 expression was significantly associated with a decreased CD8+ T cell fraction and high CD274 (encoding PD-L1) expression. These results suggest that upregulated PD-L1 inhibits CD8+ T cells, thereby causing the immune escape of cancer cells. Considerably high WHSC1L1 expression indicates worse clinical outcomes by inhibiting antitumoral immune activity. In pathway network analysis, WHSC1L1 was indirectly linked to the regulation of the lymphocyte apoptosis process.

There are few reports on specific inhibitors suitable for H3K36 methylation of the NSD family, so pharmacological inhibition of WHSC1L1 is currently not available. We suggest candidate drugs related to WHSC1L1 expression using the GDSC database, not an experimental method. We investigated 175 anticancer drugs in 50 breast cancer cell lines from the GDSC database [21]. We identified the following five anticancer drugs that can most effectively reduce the growth of breast cancer cells with high WHSC1L1 expression: VX-11e, CZC24832, LY2109761, oxaliplatin and erlotinib. VX-11e is a potent and selective ERK2 inhibitor that reduces tumor growth, proliferation and viability in a variety of cancer cell lines. VX-11e affects G0/G1 cell cycle arrest and induces high expression of p21 cell cycle inhibitors [40]. Our study showed that WHSC1L1 was indirectly linked to the regulation of the G0 to G1 transition. In liver cancer, LY2109761, a TGF-β receptor inhibitor, was shown to reduce tumor cell growth and intravascular and metastatic dissemination [41]. WHSC1L1 was also indirectly related to TGF-β, indicating that LY2109761 may be effective in breast cancer cell lines with high WHSC1L1 expression. WHSC1L1 was indirectly related to the regulation of the lymphocyte apoptotic process. PI3Kγ is related to lymphocyte activation, differentiation, and chemotaxis [42]. CZC24832, a PI3Kγ inhibitor, may be effective in breast cancer cell lines with high WHSC1L1 expression. Oxaliplatin and trastuzumab have a synergistic antitumor effect in gastric cancer cells with Erb-B2 receptor tyrosine kinase 2 (ERBB2) [43]. Our results showed that WHSC1L1 was related to the ERBB2 signaling pathway and identified specific hub genes, such as epidermal growth factor receptor (EGFR). Erlotinib could inhibit the tyrosine kinase activity of EGFR and may be a candidate drug in breast cancers with high WHSC1L1 expression [44].

This study has several limitations. First, this retrospective study had potential selection bias. Second, our study analyzed the oncogenic role of high WHSC1L1 expression using a bioinformatic approach: in silico analyses. In vivo experimental studies are needed to identify the molecular mechanisms. Third, in patients with breast cancer, the drugs suggested in this study may be different depending on disease status, microenvironments, and immunities. Further studies are needed to evaluate the therapeutic utility of WHSC1L1 inhibition in patients with breast cancer.

## 5. Conclusions

Our study revealed that WHSC1L1 was highly expressed in breast cancer tissues compared to normal tissues. High WHSC1L1 expression was associated with decreased CD8+ T cells and increased PD-L1. Pathway-based network analysis revealed a significant relationship between WHSC1L1 and the regulation of the lymphocyte apoptotic process pathway. Thus, the result could be one of several factors that can explain the relationships between high WHSC1L1 expression and low survival in patients with breast cancer. Additionally, this study confirmed the importance of WHSC1L1 in predicting survival rates using machine learning. We identified five drugs that inhibited breast cancer cells with high WHSC1L1 expression. We believe that medical oncologists and researchers will be interested in the role of WHSC1L1 in breast cancer and that our results will facilitate further studies.

## Figures and Tables

**Table 1:** Clinicopathological parameters of WHSC1L1 (Wolf-Hirschhorn syndrome candidate 1-like protein 1) in HYGH cohort.

Parameter	WHSC1L1 (HYGH Cohort)		*p*-Value
	Low (n = 224), n (%)	High (n = 232), n (%)	
Age	49.5 ± 9.3	49.5 ± 10.5	0.99 ^1^
T stage			
1	109 (48.7)	99 (42.7)	0.06 ^3^
2	109 (48.7)	117 (50.4)	
3	6 (2.7)	16 (6.9)	
N stage			
0	115 (51.3)	118 (50.9)	0.763 ^3^
1	64 (28.6)	71 (30.6)	
2	31 (13.8)	29 (12.5)	
3	14 (6.2)	14 (6.0)	
Histological grade			
1	39 (17.4)	48 (20.7)	0.335 ^3^
2	115 (51.3)	101 (43.5)	
3	70 (31.2)	83 (35.8)	
Lymphatic invasion			
Negative	123 (54.9)	107 (46.1)	0.075 ^1^
Positive	101 (45.1)	125 (53.9)	
Vascular invasion			
Negative	213 (95.1)	213 (91.8)	0.221 ^1^
Positive	11 (4.9)	19 (8.2)	
Perineural invasion			
Negative	184 (82.1)	178 (76.7)	0.189 ^1^
Positive	40 (17.9)	54 (23.3)	
ER			
Negative	53 (23.7)	76 (32.8)	**0.04** ^1^
Positive	171 (76.3)	156 (67.2)	
PR			
Negative	75 (33.5)	104 (44.8)	**0.017** ^1^
Positive	149 (66.5)	128 (55.2)	
HER2			
Negative	161 (71.9)	157 (67.7)	0.382 ^1^
Positive	63 (28.1)	75 (32.3)	
PD-L1			
Negative	155 (62.9)	127 (54.7)	**0.001** ^1^
Positive	69 (30.8)	105 (45.3)	
P53 percentage	8.4 ± 11.9	11.8 ± 13.7	**0.005** ^2^
Ki-67 index	21.5 ± 32.8	34.0 ± 38.5	**<0.001** ^2^

T or N stage, 8th edition; ER, estrogen receptor; PR, progesterone receptor; HER2, human epidermal growth factor receptor 2; PD-L1, programmed death-ligand 1; ^1^ Chi-square test; ^2^ Student’s *t*-test; ^3^ T stage: 1, 2 vs. 3; N stage: 0, 1 vs. 2, 3; Histological grade: 1, 2 vs. 3; *p* < 0.05 is shown in bold.

**Table 2:** Disease-free and disease-specific survival analyses according to WHSC1L1 (Wolf-Hirschhorn syndrome candidate 1-like protein 1) in HYGH cohort.

**Disease-Free Survival**	**Univariate ^1^**	**Multivariate ^2^**	**HR**	**95% CI**	
WHSC1L1 (low vs. high)	**<0.001**	**<0.001**	2.265	1.451	3.537
T stage (1, 2 vs. 3)	**0.001**	**0.001**	2.820	1.507	5.275
N stage (0, 1 vs. 2, 3)	**<0.001**	**0.001**	2.124	1.334	3.381
Histological grade (1, 2 vs. 3)	**0.01**	**0.05**	1.529	0.988	2.368
Lymphatic invasion (absence vs. presence)	**<0.001**	0.234	1.353	0.822	2.225
Perineural invasion (absence vs. presence)	**<0.001**	**<0.001**	2.192	1.415	3.394
Estrogen receptor (negative vs. positive)	**0.045**	**0.05**	0.647	0.414	1.011
**Disease-specific survival**	**Univariate ^1^**	**Multivariate ^2^**	**HR**	**95% CI**	
WHSC1L1 (low vs. high)	**<0.001**	**<0.001**	2.505	1.567	4.005
T stage (1, 2 vs. 3)	**<0.001**	**0.022**	2.251	1.122	4.516
N stage (0, 1 vs. 2, 3)	**<0.001**	**<0.001**	2.494	1.541	4.037
Histological grade (1, 2 vs. 3)	**0.001**	0.3	1.274	0.806	2.015
Lymphatic invasion (absence vs. presence)	**<0.001**	0.31	1.311	0.777	2.210
Perineural invasion (absence vs. presence)	**<0.001**	**<0.001**	2.477	1.586	3.868
Estrogen receptor (negative vs. positive)	**0.008**	0.076	0.655	0.410	1.046

*p* < 0.05 is shown in bold. ^1^ Log-rank test; ^2^ Cox proportional hazard model.

## Data Availability

The data presented in this study can be available on request from the corresponding author. The data are not publicly available due to privacy.
